# Immunological and nutritional perspectives on macromolecular therapies for thoracic tumors

**DOI:** 10.3389/fimmu.2025.1651482

**Published:** 2025-09-15

**Authors:** Hao Rong, Min Zheng, YunXiang Qi, Ke Ma

**Affiliations:** ^1^ Department of Thoracic Surgery, Sichuan Clinical Research Center for Cancer, Sichuan Cancer Hospital and Institute, Sichuan Cancer Center, University of Electronic Science and Technology of China, Chengdu, China; ^2^ Department of Medical Oncology, Sichuan Clinical Research Center for Cancer, Sichuan Cancer Hospital and Institute, Sichuan Cancer Center, University of Electronic Science and Technology of China, Chengdu, China; ^3^ Department of Radiation Oncology, Sichuan Clinical Research Center for Cancer, Sichuan Cancer Hospital and Institute, Sichuan Cancer Center, University of Electronic Science and Technology of China, Chengdu, China

**Keywords:** novel macromolecular drugs, thoracic tumors, mechanism of action, clinical application, efficacy evaluation, challenges and prospects

## Abstract

Thoracic tumors have high incidence and mortality rates, and present poor prognosis due to the limited efficacy of traditional therapies. Macromolecular drugs such as monoclonal antibodies and antibody-drug conjugates (ADCs) have shown promise for the treatment of lung cancer, breast cancer, and esophageal cancer. Different combinations of immunotherapy, chemotherapy, and targeted therapy have significantly improved the survival indicators of patients with thoracic tumors. Nevertheless, these combination treatment regimens have safety issues such as immune-related adverse reactions and hematological toxicity. The development of novel macromolecular drugs also faces challenges related to optimizing the affinity of antibodies, and improving the design of linkers and delivery carriers. Furthermore, the clinical application of these drugs is restricted by tumor heterogeneity, drug resistance, and exorbitant prices, along with ethical concerns and difficulties in obtaining in regulatory approval. However, macromolecular drugs present significant potential in technological innovation, combination therapy, and personalized treatment, which is expected to drive market development, improve patients’ quality of life, and reduce the socioeconomic burden of cancer. This review focuses on the application of novel macromolecular drugs for the treatment of thoracic tumors, with the aim of providing a reference for further research and clinical translation.

## 1 Introduction

### 1.1 Epidemiology of thoracic tumors

Thoracic tumors mainly include cancers of the lung, breast, esophagus, etc. The incidence and mortality rates of thoracic tumors have been increasing on an annual basis, and pose a significant threat to public health. According to the World Health Organization (WHO) (1–3), lung cancer is one of the main causes of cancer-related deaths worldwide. In 2024 alone, 2.3 million new cases of lung cancer and 1.8 million deaths were recorded globally (data from the International Agency for Research on Cancer (IARC)). In China, lung cancer ranks first in terms of both incidence and mortality among various malignancies. Furthermore, the number of young lung cancer patients is increasing year by year in China (4–6), which may be attributed to the increase in average life expectancy, exposure to air pollution, long-term smoking, and poor lifestyle habits in this demographic group (7–9).

Breast cancer is the most prevalent malignancy in women, and the number of newly diagnosed cases has been rising annually in recent years. It has become the leading cancer among Chinese women, with an increasing trend of younger onset age. The young breast cancer patients often face fertility issues after treatment due to ovarian dysfunction, and may even suffer from anxiety and depression (10–12). Almost 40% of young women with breast cancer experience varying degrees of psychological disorders during the treatment period, which seriously affects their quality of life and treatment compliance (13–15).

Esophageal cancer is relatively prevalent in some regions in Asia, and has a high fatality rate. Almost 550,000 new cases of esophageal cancer are diagnosed each year (combined with IARC data). In China, the high-incidence areas of esophageal cancer are mainly concentrated in the Henan, Hebei, Shanxi and other provinces (16–18). Esophageal cancer patients may experience symptoms such as dysphagia and malnutrition, which seriously affect their quality of life, as indicated by the significantly lower scores of physiological function, psychological state, and social function recorded by the patients compared to healthy individuals (19–21).

While conventional treatment methods like surgery, chemotherapy, and radiotherapy can improve the survival of cancer patients, the therapeutic efficacy is often limited by tumor heterogeneity, drug resistance, and damage to normal tissues (22–24). For example, orally-administered chemotherapy drugs frequently target normal hematopoietic stem cells and gastrointestinal mucosal cells, resulting in adverse reactions such as hair loss, nausea, vomiting, and decreased immunity (25–27). Moreover, due to tumor heterogeneity, individual patients have different sensitivities to chemotherapy, which can lead to drug resistance and treatment failure. Studies show that over 30% of cancer patients undergoing chemotherapy develop drug resistance during treatment (28–30).

### 1.2 Emergence of novel macromolecular drugs

Novel macromolecular drugs refer to biological agents with large molecular weight and complex three-dimensional structures, including monoclonal antibodies, bispecific antibodies, antibody-drug conjugates (ADCs), fusion proteins, and nucleic acid drugs. These agents specifically recognize and bind to antigens or specific surface markers, enabling precise ablation of target cells, distinct from traditional small-molecule chemotherapeutics (31–33). Numerous macromolecular drugs have been developed in recent years for cancer therapeutics, including monoclonal antibodies, fusion proteins, antibody-drug conjugates (ADCs) and nucleic acids. As shown in **Table 1**, antibodies with genetically engineered Fab fragment can bind to tumor antigens with high specificity and affinity (34–36), while genetic modification of the Fc segment enhances binding to the Fc receptors on immune cells, resulting in potent antibody-dependent cellular cytotoxicity (ADCC) (37–39). Furthermore, there is considerable interest in developing nanocarriers for delivering nucleic acid drugs to reduce their clearance rate and increase cellular uptake.

**Table 1:** Classification, mechanism and representative drugs.

Type	Definition	Mechanism of action	Representative drugs
Monoclonal antibodies	Highly specific antibodies produced by a single B-cell clone, containing an Fab segment and an Fc segment	Target and bind to tumor antigens, block signaling pathways; eliminate tumor cells through ADCC, CDC, and ADCP	Trastuzumab, Pembrolizumab
Bispecific antibodies	Antibodies that can simultaneously bind to two antigens or two epitopes of the same antigen	Bridge tumor cells and immune cells, activate immune killing effects	Bispecific antibodies targeting EpCAM/CD3
Antibody-drug conjugates (ADCs)	Complexes composed of monoclonal antibodies, cytotoxic drugs, and linkers	Antibodies deliver drugs to tumor cells in a targeted manner, and linkers are cleaved in the tumor microenvironment to release cytotoxic drugs	T-DM1, DS-8201, Sacituzumab govitecan
Fusion proteins	Hybrid proteins formed by the fusion of functional domains encoded by different genes	Block immunosuppressive pathways or synergistically activate immune cells	PD-1/Fc fusion protein, IFN-α/IL-2 fusion protein
Nucleic acid drugs	Including mRNA, siRNA, which function by regulating gene expression	mRNA encodes tumor antigens to activate immunity; siRNA degrades oncogene mRNA	siRNA targeting VEGF, tumor antigen mRNA vaccines

### 1.3 Aim and significance of this review

In this review, we have summarized the current status of macromolecular drugs for thoracic tumor treatment, including their mechanisms of action, clinical efficacy, safety concerns, and challenges in future development and clinical translation, which is useful for both researchers and clinicians (40–42). The PubMed and Embase databases were searched for relevant articles published in the last five years using keywords such as “novel macromolecular drugs”, “thoracic tumors”, “lung cancer”, “breast cancer”, and “esophageal cancer”, and a total of 2672 articles were obtained. The selected publications were evaluated, and 153 high-quality studies were included for the review (43–45). Literature screening was performed as per the PRISMA standard.

## 2 Mechanisms of action

### 2.1 Monoclonal antibodies

**Figure 1:**
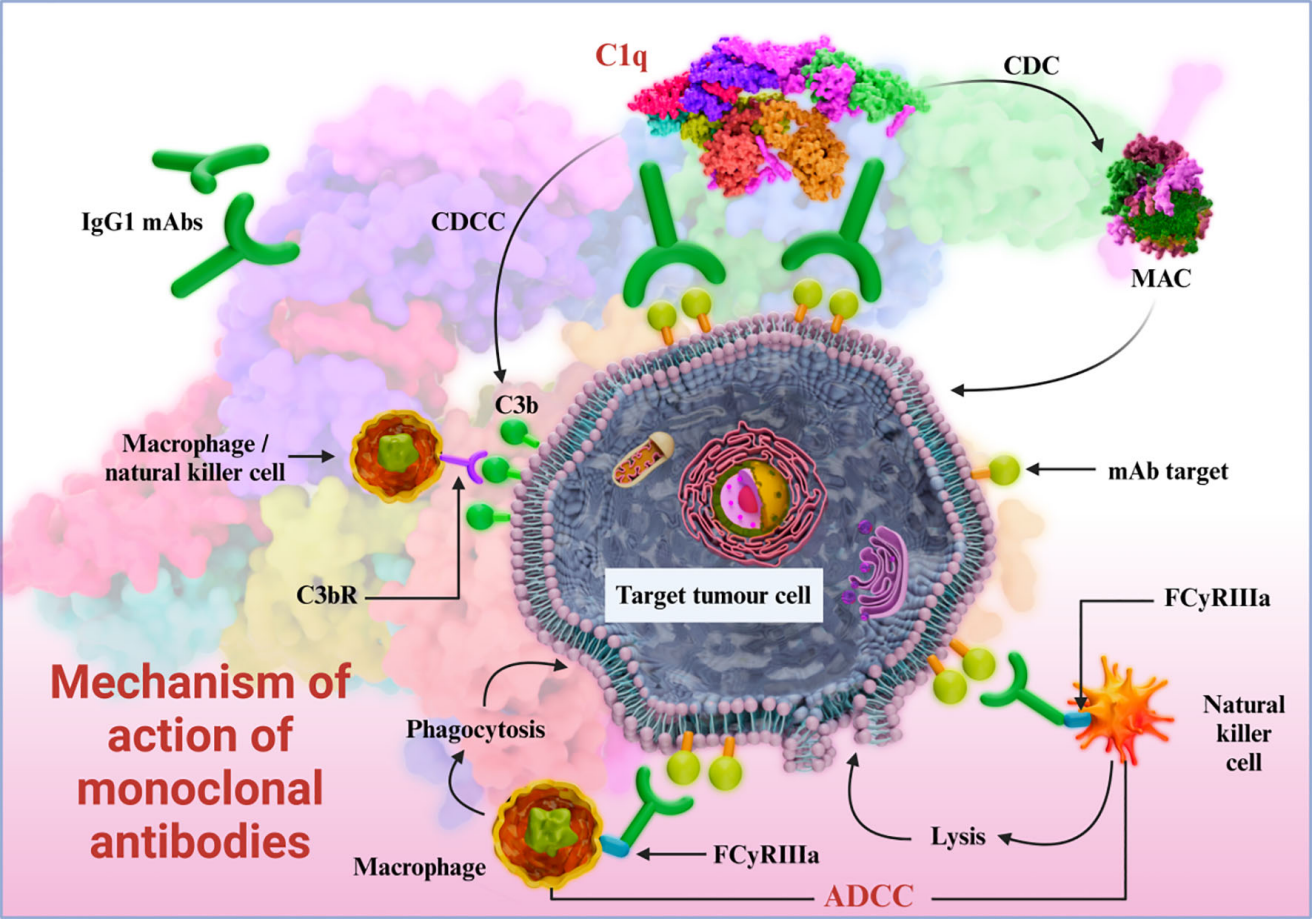
Mechanism of action of monoclonal antibodies.

Monoclonal antibodies are produced by a single B cell clone; each antibody consists of a Fab segment that binds to the cognate antigen, and a crystallizable Fc segment that mediates its effector functions through the Fc receptors expressed on immune cells (**Figure 1**). Human epidermal growth factor receptor 2 (HER2) is a transmembrane receptor tyrosine kinase that is overexpressed in some breast cancer and lung cancer cells, and promotes their growth, proliferation, invasion, and metastasis (46–48). Trastuzumab, the first monoclonal antibody approved for the treatment of HER2^+^ breast cancer, prevents autophosphorylation of HERs upon binding to its extracellular domain. The resulting blockade of the downstream signaling pathway inhibits the proliferation and survival of tumor cells. In addition, trastuzumab can also eliminate tumor cells through ADCC by recruiting natural killer (NK) cells. Bispecific antibodies contain binding sites for two different antigens or two epitopes on the same antigen. In anti - tumor applications, they simultaneously target tumor cell surface antigens and immune cell markers, forming an immunological synapse between T - cells and tumor cells. This close contact activates T - cells to secrete cytotoxic molecules and pro - inflammatory cytokines, directly killing tumor cells and enhancing immune - mediated clearance. For example, a bispecific antibody targeting EpCAM (a tumor antigen) and CD3 (a T - cell marker) has shown significant anti - tumor activity by recruiting T - cells to the tumor microenvironment and triggering their cytotoxic functions.

The activated NK cells release cytotoxic substances such as perforin and granzyme, leading to the apoptosis of tumor cells (49–51). The effector functions of an antibody are determined by its sequence and subtype (52–54). Immunotherapeutic macromolecules, a subset of novel macromolecular drugs, regulate immune responses to eliminate tumor cells, including immune checkpoint inhibitors, bispecific T-cell engagers (BiTEs), and cytokine fusion proteins. For example, monoclonal antibodies mediate immune effector functions such as: the Fc region triggers ADCC by binding to FcγRIII (CD16A) on NK cells, while interaction with serum complement molecules (C1q) induces complement-dependent cytotoxicity (CDC) via formation of a membrane-attack complex (MAC). Furthermore, the binding of Fc region with FcγRIII (CD16A), FcγRII (CD32A), and FcγRI (CD64) on macrophages triggers antibody-dependent cell-mediated phagocytosis (ADCP) (55, 56).

(57–59) Therapeutic monoclonal antibodies can also suppress tumor growth and metastasis by regulating neoangiogenesis in the tumor microenvironment (TME). For instance, the microvessel density of tumor tissues in HER2-positive (HER2^+^) breast cancer patients is significantly reduced after treatment with anti-HER2 monoclonal antibodies (57–59).

In addition to angiogenesis, the TME’s stromal components and immune cell infiltration profoundly impact antibody efficacy. Dense ECM in pancreatic ductal adenocarcinoma (PDAC) and some lung cancers acts as a physical barrier, limiting antibody penetration into tumor cores. For example, high levels of hyaluronan in TME of NSCLC reduce the accumulation of anti-PD-1 antibodies by 40-50% compared to tumors with low ECM density (preclinical models). Meanwhile, immunosuppressive cells such as tumor-associated macrophages (TAMs) and regulatory T cells (Tregs) in TME secrete cytokines that blunt ADCC-mediated tumor killing by NK cells, weakening the efficacy of trastuzumab in HER2+ breast cancer. Conversely, increased infiltration of cytotoxic T cells (CD8+) enhances the response to immune checkpoint inhibitors (ICIs), as observed in NSCLC patients with >10% CD8+ T cells in TME, who show 2.3-fold higher ORR to pembrolizumab than those with lower infiltration.

**Figure 2:**
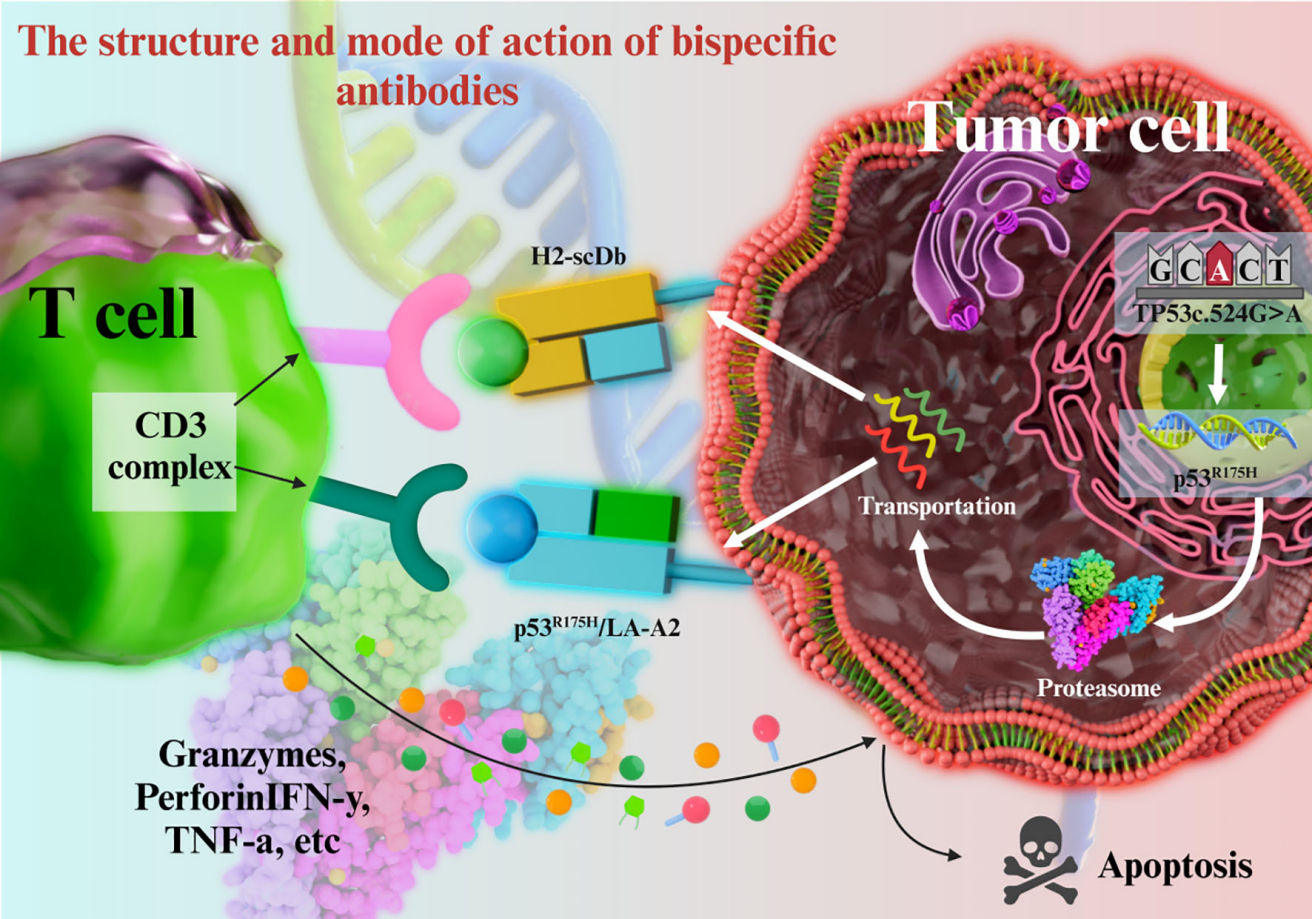
The structure and mode of action of bispecific antibodies.

Bispecific antibodies contains binding sites for two different antigens or two epitopes on the same antigen. Anti-tumor bispecific antibodies can simultaneously target antigens on the surface of tumor cells and immune cells, thus exerting a dual-pronged attack on tumor cells. For example, concurrent binding to tumor cells and T cells (via CD3) recruits the latter to the vicinity of tumor cells, resulting in the formation of an immunological synapse. The activated T cells can directly kill tumors cells by secreting cytotoxic molecules, and or enhance their immune-mediated clearance by producing cytokines such as interferon (IFN)-γ and tumor necrosis factor (TNF)-α (**Figure 2**) (60–62).

For example, a bispecific antibody that simultaneously targets the tumor-specific antigen EpCAM and CD3 molecule has shown significant anti-tumor effects (63–65). In addition, other bispecific antibodies have demonstrated therapeutic effects in some cancer patients (66–68).

### 2.2 Antibody-drug conjugates

**Figure 3:**
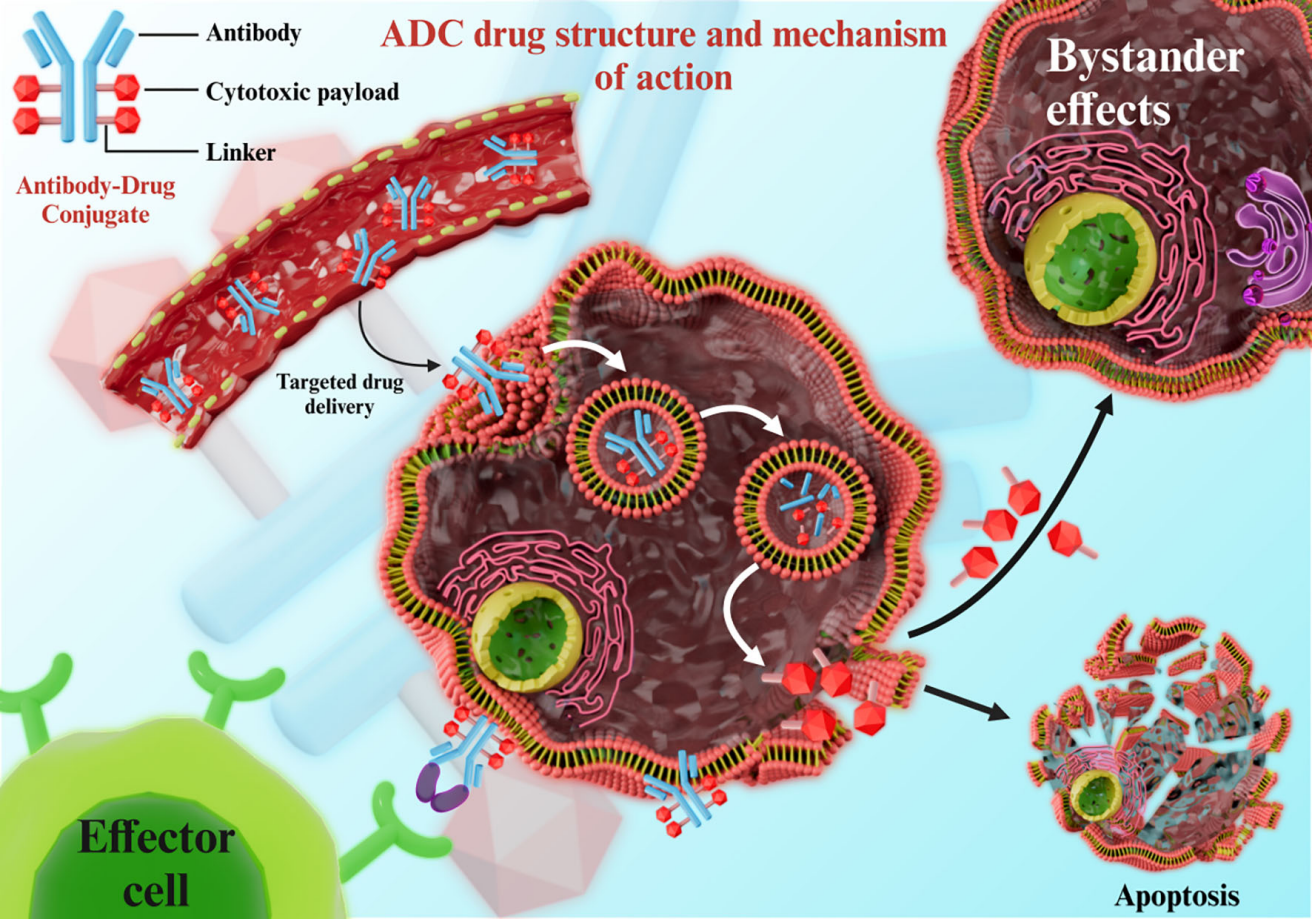
ADC drug structure and mechanism of action.

An ADC consists of a monoclonal antibody, a cytotoxic drug, and a linker. The antibody docks on the target cells through antigen binding, following which the linker is cleaved in response to specific stimuli and releases the cytotoxic drug into the diseased cells while sparing the surrounding healthy cells (**Figure 3**). ADCs achieve tumor selectivity through a three-component design: a monoclonal antibody (targeting tumor-specific antigens), a cytotoxic payload, and a linker. The antibody moiety binds to antigens overexpressed on tumor cells, enabling internalization via receptor-mediated endocytosis. Linkers remain stable in systemic circulation but are cleaved in the acidic tumor microenvironment or by tumor-specific enzymes, releasing the cytotoxic payload exclusively within tumor cells. This “targeted release” minimizes off-target toxicity to healthy tissues, enhancing therapeutic index. For instance, trastuzumab deruxtecan (DS-8201) utilizes a cleavable linker that releases its payload (DXd) preferentially in HER2-mutated NSCLC cells, achieving an ORR of 58.3% with reduced systemic toxicity.

Ado-trastuzumab emtansine (T-DM1) is conjugate of trastuzumab and the cytotoxic drug mertansine (DM1) with a cleavable thioether linker. Although trastuzumab has shown encouraging results in some HER2^+^ breast cancer patients, it is not very effective in some drug-resistant patients. T-DM1 can specifically target the HER2^+^ tumor cells through trastuzumab and deliver DMI into these cells with high precision. Once T-DMI is internalized by tumor cells, the linker is cleaved in the acidic environment of the lysosome and releases DM1, which exerts a cytotoxic effect by inhibiting tubulin polymerization and mitosis (69–74). In a large-scale clinical trial, the progression-free survival (PFS) of HER2^+^ advanced breast cancer patients treated with T-DM1 alone or in combination with chemotherapy is significantly longer than that of traditional treatment methods (75–77).

### 2.3 Fusion proteins

**Figure 4:**
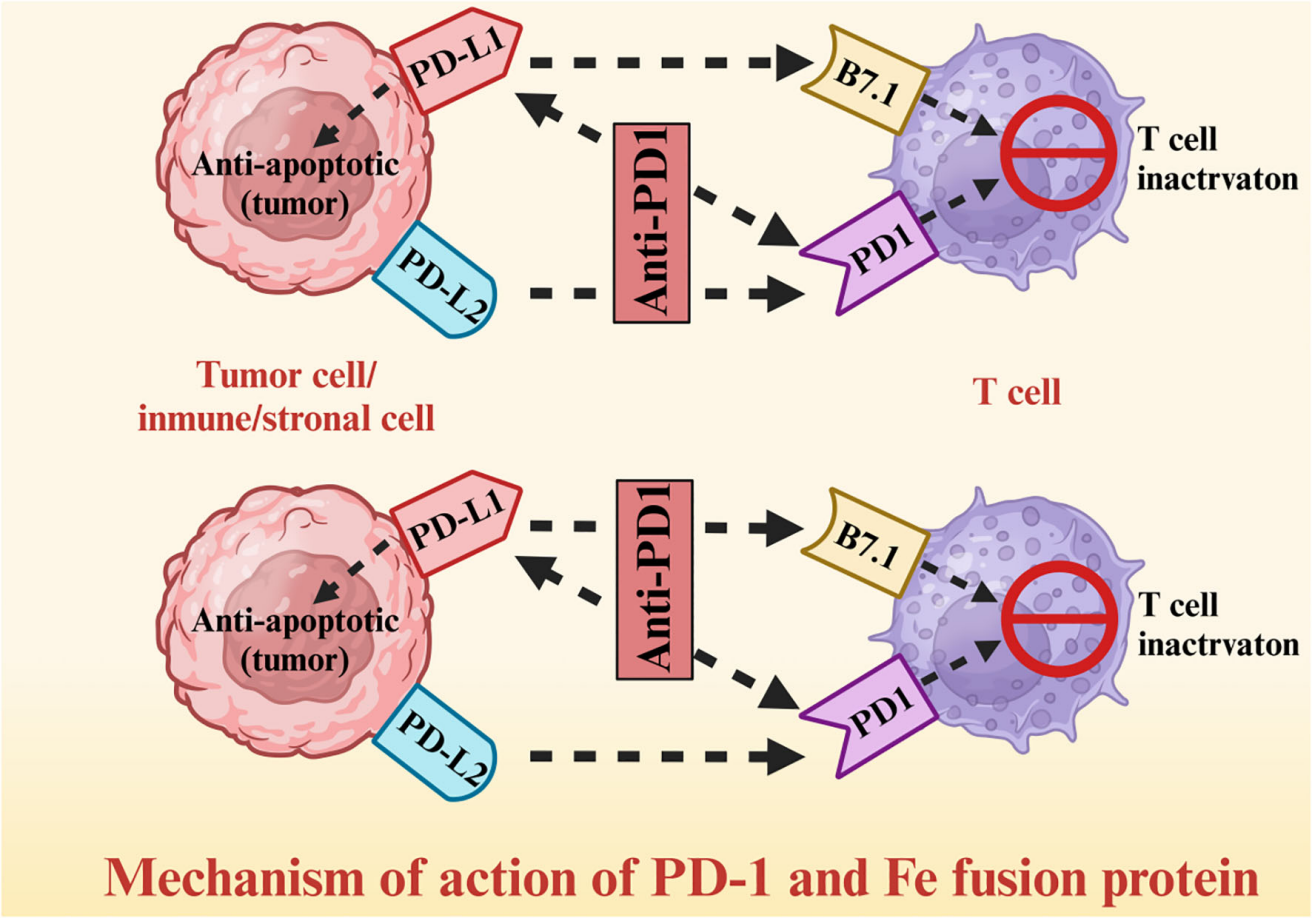
Mechanism of action of PD-1 and Fc fusion protein.

A fusion protein is a chimera of at least two domains that are encoded by distinct genes; in the context of cancer therapy, multifunctional fusion proteins have been constructed using antibody segments, cytokines, enzymes, and surface receptors. Tumor cells express high levels of the programed death receptor (PD)-1 ligand (PD - L1), which binds to PD-1 on the surface of T cells and relays inhibitory signals, thus allowing tumor cells to escape immune surveillance. A fusion construct of PD-1 and immunoglobulin Fc segment can restore T cell activity by competitively binding to PD-L1 on tumor cells and blocking the PD-1/PD-L1 signaling pathway (**Figure 4**). The PD-1/Fc fusion protein significantly improved the survival rate of non-small cell lung cancer (NSCLC) patients by activating the T cell-mediated immune response against tumor cells (78–80). The T cells released cytotoxic substances such as perforin and granzyme, and activated other immune cells by secreting interleukin (IL)-2 and IFN-γ, thus augmenting the anti-tumor immune response (81–83). Furthermore, the PD-1 fusion protein significantly enhanced the infiltration of T cells in the tumor tissues of NSCLC patients (84–86).

Hypoxia, a hallmark of TME in advanced thoracic tumors, modulates fusion protein efficacy by upregulating PD-L1 expression on tumor cells and inducing T cell exhaustion. In hypoxic regions (oxygen tension <5%), PD-L1 is stabilized via HIF-1α-mediated transcription, reducing the binding affinity of PD-1/Fc fusion proteins by 30-40% in NSCLC models. This leads to impaired T cell reactivation, as evidenced by lower IFN-γ secretion (2.1-fold reduction) compared to normoxic tumor areas. Strategies to normalize tumor vasculature may alleviate hypoxia, enhancing the efficacy of PD-1/Fc fusion proteins by improving T cell infiltration and reducing PD-L1 expression.

(81–86) Fusing multiple cytokines can potentially result in synergistic anti-tumor effects. IFN-α exerts anti-tumor and immunomodulatory effects (87–89), while IL-2 promotes the proliferation and activation of T cells and NK cells (90–92). A fusion construct of IFN-α and IL-2 significantly inhibited tumor growth in a mouse model by inducing apoptosis of tumor cells, and enhancing the anti-tumor immune response of T cells and NK cells.

### 2.4 Nucleic acid drugs

Cancer-targeting nucleic acid drugs mainly include mRNA transcripts that encode tumor-specific antigens, or non-coding RNAs that target and degrade specific mRNAs in the tumor cells. Once delivered to the tumor cells, mRNAs encoding tumor-specific antigens can be translated by the host protein synthesis machinery, resulting in increased expression of tumor antigens. The latter can then be taken up, processed, and presented by macrophages and dendritic cells to specific T cells, resulting in the T cell activation and immune-mediated clearance of tumor cells (93–95). Nucleic acid drugs rely on delivery systems to overcome instability and poor cellular uptake. Lipid nanoparticles (LNPs) and cationic polymers protect nucleic acids from nuclease degradation and facilitate endocytosis by tumor cells via electrostatic interactions with cell membranes. Targeted modification of carriers enhances accumulation in tumor tissues. For example, siRNAs targeting VEGF, encapsulated in LNPs decorated with anti-EGFR antibodies, selectively silence VEGF expression in EGFR+ esophageal cancer, inhibiting angiogenesis and tumor growth while reducing off-target effects. Exosomes, as natural nanocarriers, further improve biocompatibility and tumor penetration by leveraging cell-specific homing properties, though their clinical translation requires optimized large-scale production.

In addition to siRNA, microRNA (miRNA) mimics and antisense oligonucleotides (ASO) are also important RNA macromolecular drugs. miRNA mimics can supplement the tumor - suppressing miRNAs that are lacking in tumor cells, and inhibit tumor proliferation by targeting oncogene mRNAs. ASO can bind to specific carcinogenic RNAs, triggering their degradation or inhibiting translation. For example, ASO targeting the MYC gene has shown a tumor - shrinking effect in an esophageal cancer model. The RNA editing system based on CRISPR/Cas13 can accurately correct abnormal RNA sequences in tumor cells, and avoid the ethical risks of DNA editing. Currently, this technology has achieved specific correction of mutant RNA in lung cancer animal models, providing a new strategy for unresectable tumors.

Despite advantages such as short development cycles and versatility, mRNAs are easily degraded by nucleases, and cannot penetrate cell membranes easily die to a net negative charge. The siRNA drugs can inhibit oncogene expression in tumor cells by degrading the target mRNAs. The siRNAs form double-strand structures with the specific mRNA by complementary pairing, resulting in the activation of intracellular nucleases such as Dicer enzyme and RNA-induced silencing complex (RISC). For example, siRNA drugs targeting vascular endothelial growth factor (VEGF) can inhibit tumor growth and metastasis by blocking angiogenesis in the TME. However, the *in vivo* delivery and stability of siRNAs remain the key limiting factors for their clinical application (22, 96, 97), since siRNAs are also easily degraded by nucleases (98). Lipid nanoparticles and cationic polymers can be used to improve the transport and stability of nucleic acid drugs, although these carriers also have limitations pertaining to biocompatibility, targeting ability, and delivery efficiency. Currently, researchers are actively exploring various delivery strategies, such as nanoscale delivery systems and modification of the carriers with targeting ligands. The efficacy of exosomes as delivery carriers for siRNA drugs has been tested in animal models, and the results are encouraging.

## 3 Current progress

### 3.1 Lung cancer

The combination of immune checkpoint inhibitors (ICIs) and chemotherapy has replaced single-drug chemotherapy as the standard first-line treatment for advanced NSCLC. In the KEYNOTE-189 study, pembrolizumab combined with pemetrexed and platinum-based chemotherapy significantly improved outcomes in advanced non-squamous NSCLC patients: the median PFS was extended from 4.9 months to 8.8 months, and the median OS was prolonged from 11.3 months to 19.2 months. Notably, the 2-year OS rate in the combination group was 45.7% versus 27.3% in the chemotherapy group, confirming long-term survival benefits.

Similarly, the IMpower130 trial demonstrated that atezolizumab plus carboplatin and nab-paclitaxel significantly improved median OS and PFS in advanced non-squamous NSCLC patients, including those with EGFR/ALK mutations. The ORR in the combination group was 49% compared to 32% with chemotherapy alone, highlighting the synergistic effect of immunotherapy and chemotherapy. In addition, the combination of targeted therapy and chemotherapy has also achieved good efficacy in some NSCLC patients with epidermal growth factor receptor (EGFR) mutations or anaplastic lymphoma kinase (ALK) fusions. Some new ADC drugs, such as trastuzumab deruxtecan (99–101), have achieved an objective response rate (ORR) of 58.3% in NSCLC cancer with HER2 mutations.

In the IMpower110 trial (NCT02409342), atezolizumab monotherapy demonstrated superior overall survival (OS) compared to chemotherapy in PD-L1-positive NSCLC patients with high expression (TC ≥50% or IC ≥10%), with a median OS of 20.2 months versus 13.1 months, supporting its role as a first-line option for selected patients.

First-generation EGFR-targeting tyrosine kinase inhibitors (TKIs), such as gefitinib and erlotinib, have achieved good efficacy in NSCLC patients with EGFR mutations and extended their PFS. However, most patients develop drug resistance within a short duration due to secondary mutations in the EGFR gene, such as the T790M mutation. The second-generation EGFR-TKI afatinib has been effective in some drug-resistant patients, but shows limited efficacy in others (102–104). Osimertinib, a third-generation EGFR-TKI, is not only effective in newly diagnosed EGFR-mutated NSCLC patients, but has also prolonged the PFS and OS of drug-resistant patients with the T790M mutation. In the phase III FLAURA trial, osimertinib achieved a median PFS duration of 18.9 months in EGFR-mutated NSCLC patients as the first-line treatment.

The CheckMate 227 trial (NCT02477826) further validated the efficacy of dual immunotherapy: nivolumab plus ipilimumab significantly improved OS in NSCLC patients with high tumor mutational burden (TMB ≥10 mut/Mb) compared to chemotherapy, establishing a chemotherapy-free option for this subgroup.

ALK fusion^+^ NSCLC patients also respond well to ALK inhibitors such as crizotinib, ceritinib, and alectinib. The first-generation ALK inhibitor crizotinib can significantly improve the survival of ALK fusion^+^ patients, although some may develop resistance due to secondary mutations or bypass activation of the ALK gene (105–107). The second-generation ALK inhibitors ceritinib and alectinib have shown good therapeutic efficacy in patients with crizotinib-induced drug resistance. In addition, alectinib is more effective in first-line treatment for NSCLC, and has prolonged the PFS and OS of patients. In a global multicenter clinical trial (ALEX), alectinib extended the median PFS of patients to 34.8 months.

(108–110) SCLC is a highly malignant type of lung cancer that 'is relatively sensitive to chemotherapy and radiotherapy, but prone to recurrence and metastasis. It is currently treated with etoposide and platinum-based chemotherapy and concurrent chemoradiotherapy, although the 5-year survival rate is very low (111–113). Immune checkpoint inhibitors (ICIs) such as durvalumab (anti-PD-1), atezolizumab (anti-PD-L1), and nivolumab (anti-PD-1) have improved SCLC outcomes. For example, durvalumab consolidation after chemoradiotherapy in the PACIFIC trial extended PFS from 5.6 to 16.8 months and OS from 28.7 to 47.5 months (108–110). Moreover, combination regimens of immunotherapy, chemotherapy and radiotherapy are continuously being explored to further improve the outcomes of SCLC patients. A clinical trial investigating the efficacy of atezolizumab combined with chemotherapy as first-line treatment for SCLC is currently underway, and the inityial results are encouraging (114–116).

### 3.2 Breast cancer

HER2^+^ breast cancer accounts for about 15-20% of all breast cancers, and is characterized by invasiveness and a high risk of recurrence (117–119). Large-scale clinical studies have shown that the combination of trastuzumab and chemotherapy can increase the 5-year survival rate of HER2^+^ breast cancer patients to about 80%. Targeted biological agents, such as trastuzumab, specifically recognize overexpressed antigens on tumor cells or aberrant signaling pathways, exerting anti-tumor effects with minimal toxicity to normal tissues (117–119). Like trastuzumab, pertuzumab also targets the extracellular domain of the HER2 receptor, albeit at a different site. Therefore, the combination of both drugs can synergistically block the HER2 signaling pathway (120–122). In the multicenter CLEOPATRA trial, this dual-target combination prolonged the PFS of HER2^+^ breast cancer patients compared to those in the single-drug groups, and reduced the risk of disease recurrence. The conventional drugs for HER^+^ breast cancer are ineffective in patients with brain metastases due to their inability to cross the blood-brain barrier. DS-8201, an ADC consisting of trastuzumab and a topoisomerase I inhibitor attached with a cleavable linker, can penetrate the blood-brain barrier due to its structure (123–125). In the DESTINY-BC01 trial, DS-8201 significantly reduced the intracranial tumors in some HER^+^ breast cancer patients with brain metastases, and achieved an ORR of 64%.

Triple-negative breast cancer (TNBC) cells do not express estrogen receptors (ER), progesterone receptors (PR), or HER2. Therefore, endocrine therapy and HER2-targeted therapy are ineffective in TNBC patients, resulting in poor prognosis. Pembrolizumab, an anti-PD-1 monoclonal antibody, shows certain efficacy in PD-L1^+^ TNBC patients. In a phase III clinical study (KEYNOTE-355 study), the combination of pembrolizumab and chemotherapy significantly increased the ORR and PFS of PD-L1^+^ TNBC patients compared to chemotherapy alone. The ORR of the combination treatment group reached 21% - 35% and the PFS was extended by 1.5–2 months, with certain differences between the subgroups. Sacituzumab govitecan is an ADC drug that targets Trop-2, a glycoprotein overexpressed on TNBC cells, and induces a cytotoxic effect through the topoisomerase-I inhibitor SN-38 (derivative of irinotecan). In the ASCENT study, Sacituzumab govitecan monotherapy achieved an ORR of 35% in heavily pretreated TNBC patients, with a median PFS of 4.8 months and median OS of 11.8 months, significantly superior to chemotherapy. For PD-L1-positive TNBC, the KEYNOTE-355 trial showed that pembrolizumab plus chemotherapy improved median PFS from 5.6 months to 7.5 months in patients with a CPS ≥10. The ORR was 53% in the combination group versus 40% in the chemotherapy group, with a 2-year OS rate of 35% versus 22%, confirming the value of immunochemotherapy in this subgroup (126–128).

### 3.3 Esophageal cancer

Traditional chemotherapy regimens can relieve the symptoms of esopahgeal cancer without providing any significant survival benefit for patients. The combination of nivolumab and chemotherapy as first-line treatment can improve the quality of life of patients with advanced esophageal cancer. In one clinical trial, nivolumab combined with chemotherapy extended the OS of esophageal cancer patients to 13.2 months compared to 11.1 months in the traditional chemotherapy group (reported in the CheckMate-648 study (NCT03143153)). Fibroblast growth factor receptor (FGFR) is expressed at aberrantly high levels in esophageal cancer, suggesting its potential as a therapeutic target. Indeed, small molecule drugs and monoclonal antibodies targeting FGFR have been shown to inhibit proliferation, migration, and angiogenesis in esophageal cancer by blocking the FGFR signaling pathway (129–131), and are currently in the clinical testing phase. In an early-stage clinical trial, FGFR inhibitor monotherapy achieved tumor control in some FGFR^+^ esophageal cancer patients, indicating good application prospects.

## 4 Clinical efficacy and safety evaluation

### 4.1 Efficacy evaluation indicators

The ORR refers to the proportion of patients with tumor shrinkage that meets certain criteria, and is an important indicator for evaluating the short-term efficacy of anti-cancer drugs. Multiple clinical trials have shown that the ORR of combination treatment regimens using novel macromolecular drugs is higher than that of traditional single-drug treatments. For example, the combination of pembrolizumab and chemotherapy increased the ORR of NSCLC patients by 20% compared with chemotherapy alone (KEYNOTE-189). In the IMpower150 trial, atezolizumab plus bevacizumab, carboplatin, and paclitaxel achieved an ORR of 63% in advanced non-squamous NSCLC, compared with 48% in the bevacizumab-containing chemotherapy group, with a median OS extension of 4.5 months (132–134).

Immune checkpoint inhibitors (ICIs) activate T cells to recognize and attack tumors, synergizing with chemotherapy to enhance clearance. Their continuous immune activation delays recurrence, prolonging PFS and OS.

The efficacy indices of the different treatment regimens are summarized in **Table 2**.

**Table 2:** Efficacy of different treatment regimens.

Tumor type	Treatment regimen	Objective response rate (ORR)	Disease control rate (DCR)	Progression - free survival (PFS, months)	Overall survival (OS, months)
Non - Small - Cell Lung Cancer	Pembrolizumab + Pemetrexed + Platinum - based Chemotherapy	47.6%	76.4%	8.8	19.2
Non - Small - Cell Lung Cancer	Traditional Chemotherapy (Pemetrexed + Platinum - based)	18.9%	49.4%	4.9	11.3
Non - Small - Cell Lung Cancer (EGFR - Mutated)	Osimertinib as First - Line Treatment	71%	93%	18.9	Immature
Non - Small - Cell Lung Cancer (ALK - Fusion - Positive)	Alectinib as First - Line Treatment	72.5%	96.2%	34.8	Not Reached
Non - Small - Cell Lung Cancer (HER2 - Mutated)	Trastuzumab Deruxtecan Treatment	58.3%	81.3%	8.2	17.8
Small - Cell Lung Cancer	Durvalumab in Consolidation Treatment after Concurrent Chemoradiotherapy	28.4%	66.3%	16.8	47.5
Small - Cell Lung Cancer	Traditional Concurrent Chemoradiotherapy (Etoposide + Platinum - based)	16.0%	41.0%	5.6	28.7
HER2 - Positive Breast Cancer	Trastuzumab + Chemotherapy	75%	89%	No Specific Single Data	No Specific Single Data
HER2 - Positive Breast Cancer	Dual - Target Combination of Pertuzumab and Trastuzumab	80.2%	93.8%	18.5	56.5
HER2 - Positive Breast Cancer (Brain Metastases)	DS - 8201 Treatment	64%	No Specific Single Data	No Specific Single Data	No Specific Single Data
Triple - Negative Breast Cancer	Sacituzumab Govitecan Monotherapy	35%	46%	4.8	11.8
Triple - Negative Breast Cancer (PD - L1 - Positive)	Pembrolizumab + Chemotherapy (Albumin - Bound Paclitaxel)	21% - 35%	No Specific Single Data	5.6	No Specific Single Data
Esophageal Cancer	Nivolumab + Chemotherapy (Fluorouracil + Platinum - based)	45.9%	72.5%	7.7	13.2
Esophageal Cancer	Traditional Chemotherapy (Fluorouracil + Platinum - based)	29.4%	55.7%	5.6	11.1

### 4.2 Comparison with traditional treatment methods

Macromolecular drugs exhibit distinct advantages and limitations compared to traditional small-molecule therapies in thoracic tumor treatment: Advantages of macromolecular drugs:

Monoclonal antibodies, ADCs, and fusion proteins target tumor-specific antigens or receptors, minimizing off-target effects on normal tissues. For example, trastuzumab specifically binds HER2+ breast cancer cells, whereas small-molecule EGFR-TKIs may inhibit EGFR in normal epithelia, causing skin rash or diarrhea. Due to larger molecular size and slower clearance, macromolecular drugs often have longer half-lives (days to weeks), reducing administration frequency compared to small molecules. Bispecific antibodies and fusion proteins simultaneously modulate multiple pathways, whereas small molecules typically act on single targets.

Limitations of macromolecular drugs: Large molecular weight hinders penetration into solid tumors with dense stroma, whereas small molecules diffuse more freely. Most macromolecular drugs require intravenous or subcutaneous injection, unlike oral small molecules, reducing patient convenience. Antibody engineering, ADC linker synthesis, and nucleic acid delivery systems are more expensive to manufacture, leading to higher drug prices compared to small molecules.

For instance, the combination of trastuzumab and chemotherapy showed better efficacy and fewer side effects for HER2+ breast cancer patients compared to chemotherapy alone. This can be attributed to the specific targeting of the HER+ tumor cells by trastuzumab, which minimizes damage to normal cells. In contrast, chemotherapy drugs also exert cytotoxic effects on other cycling cells, such as hematopoietic stem cells, gastrointestinal mucosal cells, etc., resulting in hair loss, nausea, vomiting, compromised immunity, and other adverse reactions (135–137). Furthermore, ICIs and chemotherapy drugs also act synergistically to eliminate tumors and improve clinical outcomes. Chemotherapy disrupts the tumor structure and facilitates release of tumor-associated antigens, which are subsequently recognized by the T cells and trigger a potent immune response. On the other hand, the ICIs can augment the cytotoxic effects of chemotherapeutic drugs by modulating the TME, and achieve long-term tumor control by relieving tumor-induced immune suppression and activating the endogenous anti-tumor immune response.

**Figure 5:**
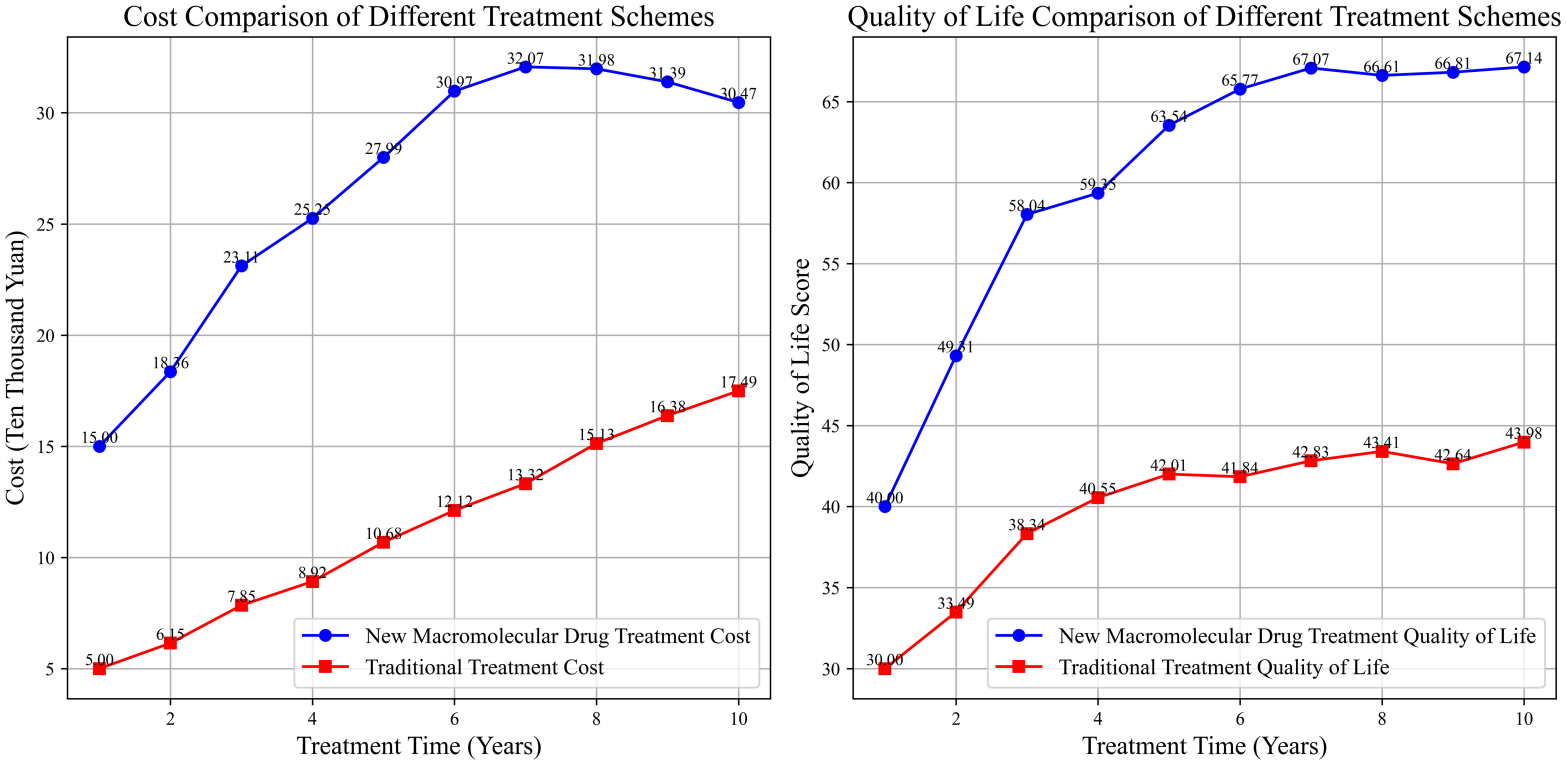
Comparison of the cost-effectiveness of different treatment options.

Although macromolecular drugs are relatively expensive owing to the high costs of development and production, they may be cost-effective in the long run considering the extended survival of patients, improved quality of life, and the reduction in subsequent treatment costs caused by tumor recurrence and metastasis. The combination of ICIs and chemotherapy can not only extend the survival period of patients but also reduce the costs of re-surgery, chemotherapy, and other treatments caused by tumor recurrence. In addition, patients can maintain a relatively high quality of life during the survival period, which may result in economic benefits. For instance, lung cancer patients treated with ICIs and chemotherapy face high initial treatment costs, but the overall cost within a 5-year survival period is equivalent to that of traditional multiple-round chemotherapy, and the patients have a higher quality of life (138–140). The cost-effectiveness curves of different treatment regimens are shown in **Figure 5**.

The adverse reactions associated with traditional chemotherapy, such as nausea, vomiting, and hair loss, seriously affect patients’ lives. Given the high specificity and fewer adverse reactions of macromolecular drugs, the patients can maintain better physical functions and a more positive psychological state during the treatment period. For example, the physical function scores of breast cancer patients who receive targeted therapy are 8–15 points higher (out of 100) than that of patients receiving only chemotherapy (141–143). Furthermore, patients can engage in more daily activities such as walking and doing housework during the targeted therapy regimen, and are therefore less likely to experience negative emotions such as anxiety and depression, resulting in better treatment compliance. Novel macromolecular drugs also have certain advantages in terms of patient convenience. For instance, antibody drugs are usually administered through subcutaneous injections, and therefore require fewer hospital visits and shorter hospital stays compared to intravenous chemotherapy. In addition, some of these subcutaneously-injectable antibody drugs can be self-administered by patients at home, which significantly improves treatment compliance. However, some nucleic acid drugs need complex delivery systems, and also have stringent requirements for storage temperature, humidity, and other environmental conditions, which limits their application in centers with few resources (144–146).

### 4.3 Safety and adverse reactions

ICIs can trigger pneumonitis, enteritis, thyroiditis, and other inflammatory reactions due to the over-activation of the immune system, thereby warranting close monitoring and timely treatment. The incidence of immune-related adverse reactions in NSCLC patients undergoing immunotherapy is approximately 5%. Most of the symptoms can be relieved in a timely manner with glucocorticoids and other anti-inflammatory medications. Immune-related adverse events (irAEs) can range from mild to life-threatening. Severe cases of immune-mediated pneumonitis occur in 1–3% of NSCLC patients treated with ICIs, requiring high-dose corticosteroids or immunosuppressants. Colitis, another common irAE, affects 2–5% of patients, presenting with diarrhea and abdominal pain, which may progress to intestinal perforation if untreated. Endocrine toxicities, such as hypothyroidism and adrenal insufficiency (1–2%), often require lifelong hormone replacement. Notably, irAEs may persist or recur after treatment discontinuation, necessitating long-term monitoring.

ADCs can cause hematological toxicity (such as anemia, thrombocytopenia, etc.) due to the adverse effects of the drug component on hematopoietic stem cells, along with liver and kidney damage, and infusion-related reactions and skin allergies. The incidence of hematological toxicity in patients treated with T-DM1 is 50% - 70%, and that of anemia and thrombocytopenia are 40% - 60% and 20% - 40% respectively (specific hematological toxicity indicators and relevant research need to be filled in here). Hematological toxicity can be alleviated by adjusting the drug dose and through supportive treatment such as blood transfusion, erythropoietin infusion, etc.

The adverse reactions of nucleic acid drugs are mainly associated with the immunogenicity of the carriers, and include inflammatory reactions at the injection site, allergic reactions, etc. These drugs may also affect the function of normal cells by silencing off-target mRNAs through non-specific binding. Clinical trials of mRNA vaccines have reported 0-15% incidence rate of inflammatory reactions at the injection site, and reactions are mostly mild and moderate (147–149). The adverse reactions of various novel macromolecule drugs are summarized in **Table 3**.

**Table 3:** Adverse reactions of novel macromolecular drugs.

Drug type	Specific drug	Adverse reaction	Incidence rate
Immune Checkpoint Inhibitor	Pembrolizumab	Immune - related Pneumonitis	5% in non - small - cell lung cancer immunotherapy
Immune Checkpoint Inhibitor	Pembrolizumab	Immune - related Pneumonitis	3% - 10%
Immune Checkpoint Inhibitor	Pembrolizumab	Immune - related Thyroiditis	5% - 15%
Immune Checkpoint Inhibitor	Nivolumab	Immune - related Pneumonitis	Approximately 4% - 8% in non - small - cell lung cancer treatment
Immune Checkpoint Inhibitor	Nivolumab	Immune - related Pneumonitis	2% - 8%
Immune Checkpoint Inhibitor	Nivolumab	Immune - related Thyroiditis	6% - 12%
ADC Drug	Ado - trastuzumab Emtansine (T - DM1)	Hematological Toxicity (Anemia)	40% - 60%
ADC Drug	Ado - trastuzumab Emtansine (T - DM1)	Hematological Toxicity (Thrombocytopenia)	20% - 40%
ADC Drug	Ado - trastuzumab Emtansine (T - DM1)	Liver and Kidney Function Damage	Hepatic Toxicity (Elevated Transaminases) approximately 10% - 20%; Renal Function Damage is relatively rare, 5% - 10%
ADC Drug	Ado - trastuzumab Emtansine (T - DM1)	Infusion - related Reaction	10% - 30%
ADC Drug	Ado - trastuzumab Emtansine (T - DM1)	Skin Toxicity	20% - 40%
ADC Drug	Sacituzumab Govitecan	Hematological Toxicity (Neutropenia)	50% - 70%
ADC Drug	Sacituzumab Govitecan	Diarrhea	30% - 50%
Nucleic Acid Drug (mRNA Drug)	Various mRNA Tumor - treating Drugs	Inflammatory Reaction at the Injection Site	10% - 30%
Nucleic Acid Drug (mRNA Drug)	Various mRNA Tumor - treating Drugs	Allergic Reaction	5% - 10%
Nucleic Acid Drug (mRNA Drug)	Various mRNA Tumor - treating Drugs	Off - target Effect	–
Nucleic Acid Drug (mRNA Drug)	siRNA Drug Targeting VEGF	Inflammatory Reaction at the Injection Site	10% - 20%
Nucleic Acid Drug (mRNA Drug)	siRNA Drug Targeting VEGF (Under Research)	Allergic Reaction	5% - 10%
Nucleic Acid Drug (mRNA Drug)	siRNA Drug Targeting VEGF (Under Research)	Off - target Effect	–

### 4.4 Regulatory approvals of macromolecular drugs

Several novel macromolecular drugs have obtained regulatory approvals from global authorities for thoracic tumor treatment. For example, the FDA has approved pembrolizumab (anti-PD-1) for first-line treatment of advanced NSCLC in combination with chemotherapy, based on the KEYNOTE-189 trial demonstrating improved OS and PFS. DS-8201 received accelerated approval by the FDA for HER2-mutated NSCLC due to its 58.3% ORR in clinical trials.

In the EU, the EMA has authorized atezolizumab in combination with chemotherapy for advanced non-squamous NSCLC (IMpower130 trial) and durvalumab as consolidation therapy after chemoradiotherapy for stage III NSCLC (PACIFIC trial). For breast cancer, T-DM1 was approved by both FDA and EMA for HER2+ advanced breast cancer, showing prolonged PFS compared to traditional regimens.

In China, NMPA has approved trastuzumab for HER2+ breast cancer and nivolumab in combination with chemotherapy for advanced esophageal cancer. These approvals validate the clinical value of macromolecular drugs, while ongoing post-marketing surveillance ensures long-term safety.

## 5 Challenges and limitations

### 5.1 Research and development

The anti-tumor effect of antibody drugs depends on their affinity for the tumor-specific antigens. However, optimizing the affinity of antibody drugs may affect their structural stability and specificity. Some researchers are currently using computer-aided design, directed evolution, and other technologies to improve the affinity antibody drugs without affecting their stability. Computer-aided design predicts the impact of different amino acid mutations on affinity by simulating the antibody-antigen binding mode, thus enabling precise antibody modification. On the other hand, directed evolution mimics the process of natural evolution, which can be useful for random mutagenesis and screening *in vitro*. However, introducing mutations or other variations may cause changes in antibody structure and affect their *in vivo* pharmacokinetic properties.

Besides delivery issues, RNA is easily degraded by intracellular RNases, and long-term expression may trigger immunogenicity. Novel chemical modifications can increase the half-life of siRNA to more than 72 hours, but this will increase the synthesis cost. Deglycosylation modification may lead to changes in the pharmacokinetics of antibodies. Some deglycosylated ADC drugs exhibit an increase in hepatotoxicity, and it is necessary to optimize the modification sites through computer simulation.

The safety and efficacy of ADC drugs depend on the stability and cleavability of the linkers. Unstable linkers may lead to the premature release of cytotoxic drugs in circulation, resulting in serious side effects. On the other hand, uncleavable linkers can impair the therapeutic effect of ADCs by preventing the release of cytotoxic drugs in the tumor cells. Enzyme-responsive and pH-responsive linkers are cleaved in the TME due to the presence of specific enzymes or under acidic conditions, and release the cytotoxic drug at the tumor site. This targeted drug release can not only improve the specificity and efficacy of ADCs but also reduce damage to normal tissues. However, the synthesis of such novel linkers is a complex process, resulting in higher production costs. Moreover, the *in vivo* stability and cleavage efficiency of these linkers still need to be optimized.

The effective delivery of nucleic acid drugs has always been a bottleneck in their clinical application. Although lipid nanoparticles and cationic polymers have improved the efficiency of delivering nucleic acid drugs, the poor biocompatibility and low targeting ability of these carriers remain significant challenges in clinical applications. Exosomes are nanoscale vesicles that are secreted by various cells, and exhibit good biocompatibility and low immunogenicity, making them suitable drug carriers. However, large-scale preparation of exosomes is hampered by low yields and high costs, and targeted modification is a pressing issue that remains to be solved. In addition to delivery efficiency, macromolecular drugs face critical barriers in tissue penetration and tumor vascular permeability. The dense extracellular matrix (ECM) of solid tumors, rich in collagen and hyaluronan, forms a physical barrier that hinders antibody penetration, limiting their access to deep-seated tumor cells. For example, monoclonal antibodies with molecular weights exceeding 150 kDa exhibit poor diffusion through the ECM, resulting in uneven distribution within tumor masses. Tumor vascular hyperpermeability, while exploited by the enhanced permeability and retention (EPR) effect, is often disorganized and leaky, leading to inconsistent drug accumulation. Strategies to improve penetration include conjugation with ECM-degrading enzymes or engineering smaller antibody fragments with reduced molecular size, which have shown 2–3 fold higher tumor penetration in preclinical models.

### 5.2 Clinical translation

Due to the significant genetic, proteomic, and biological heterogeneity of thoracic tumors, may patients do not respond to novel macromolecular drugs. It is challenging to screen patients who can benefit from these treatments, or sensitize the drug-resistant tumor cells. Several research groups are employing the multi-omics approach, including genomics, transcriptomics, and proteomics, to identify biomarkers related to drug sensitivity, enable patient stratification, and help devise individualized treatment strategies. Multi-omics analyses can elucidate the mutation landscape (genomics), gene expression patterns (transcriptomics), and protein expression and modifications (proteomics) in tumor cells, thus provided an integrated molecular picture. However, multi-omics data analysis is relatively complex, and requires trained professionals along with high-performance computers. Moreover, there are still many difficulties in the clinical verification and translation of biomarkers identified using this approach. Emerging drug resistance significantly hinders the long-term efficacy of novel macromolecular drugs. For example, HER2-targeted antibodies and ADCs often face acquired resistance due to HER2 gene mutations, activation of bypass signaling pathways, or reduced antigen expression. In NSCLC, EGFR-TKI resistance occurs in over 50% of patients within 12–18 months, driven by T790M mutations or MET amplification. Similarly, PD-1/PD-L1 inhibitors may fail due to loss of HLA expression or upregulation of alternative immune checkpoints. Overcoming resistance requires combinatorial strategies, such as co-targeting resistance pathways or developing next-generation agents with broader epitope coverage.

Reports of drug resistance are gradually emerging as more and more of these macromolecular drugs enter clinical application. Tumor cells develop drug resistance through multiple mechanisms, such as target mutations and compensatory activation of signaling pathways. A critical challenge in cancer treatment is to elucidate the mechanisms of resistance to specific drugs and develop strategies to overcome drug resistance. Resistance to HER2-targeted therapies can be attributed to mutations in the HER2 gene, activation of downstream signaling pathways, and changes in the TME. Thus, combining drugs with different mechanisms of action, such as inhibitors of the PI3K/AKT/mTOR signaling pathway or immunotherapy drugs, may be effective against recalcitrant HER2^+^ tumors. However, these combination therapy regimens need further optimization and safety evaluation.

Macromolecular drugs are costly due to the considerable investment in research and development. This limits their widespread clinical application and is not conducive to solving the unreasonable distribution of medical resources. Government policies, centralized procurement, and other means are required to improve drug accessibility. For instance, tax incentives to pharmaceutical companies can reduce the costs of research and development, and even encourage production of generic drugs. In addition, centralized procurement can reduce the purchase price of drugs through the scale effect. Furthermore, generic drugs can increase market competition and promote price reduction of original drugs. However, the implementation of these measures still faces many difficulties, such as balancing the interests of pharmaceutical companies and quality control of generic drugs.

### 5.3 Supervision and ethics

The research, development, and marketing of novel macromolecular drugs need to go through strict regulatory approval procedures, including aspects such as clinical trial design, implementation, and data monitoring. While this would ensure the safety and effectiveness of the drugs, it will also extend the research and development cycle, as well as the marketing time. To accelerate drug development, regulatory authorities need to explore more flexible and efficient approval procedures, such as breakthrough therapy designation and priority review, and strengthen the guidance and supervision of clinical trials to ensure the quality and safety of drugs. Breakthrough therapy designation and priority review can expedite the approval process of drugs with obvious clinical advantages. However, the quality and safety of drugs have to be ensured when accelerating the approval process in order to avoid potential risks. Furthermore, it is necessary to fully consider ethical and legal issues concerning the application of macromolecular drugs, and formulate strict norms and guidelines. For instance, there are ethical concerns regarding mRNAs and siRNAs due to the irreversibility of gene editing and the potential impact on human germ cells. Multiple international ethical committees have been established to supervise the research on gene therapy, clarify the scope of application and limitations of gene editing, and ensure the safety and ethical rationality of such therapies.

## 6 Future directions

### 6.1 Trends in technological innovation

Artificial intelligence (AI) and machine learning technologies can be used to design and screen antibodies with higher affinity and specificity, and improve the efficacy of antibody-based drugs. Novel antibodies designed using AI technology have already entered clinical testing. AI can predict the affinity and specificity of novel antibodies by analyzing antibody structure and function data, thereby accelerating research and development. Furthermore, incorporation of humanized sequences in monoclonal antibodies can improve their safety by reducing immunogenicity, while glycosylation modification can alter the pharmacokinetic properties of antibodies and enhance binding to targets. Developing ADCs that can intelligently respond to the characteristics of the TME, such as low pH, enzyme activity, etc., can improve drug efficacy and reduce side effects by ensuring targeted delivery and release. For instance, a pH-responsive linker can achieve efficient release of cytotoxic drugs within the acidic TME while minimizing damage to normal tissues. Likewise, development of linkers responsive to other conditions in the TME, such as temperature and redox potential, can further improve the efficacy of ADC drugs. The delivery and efficacy of nucleic acid drugs can be significantly improved by using carriers based on exosomes, nanobodies, and viral vectors. As natural nanovesicles, exosomes have low immunogenicity and good biocompatibility, and are ideal tools for drug delivery. Nanobodies have the advantages of low molecular weight, high specificity, and stability, and may improve the targeting ability of drug carriers. Viral vectors have the ability of efficient gene transduction and can precisely deliver nucleic acid drugs to target cells. The combination of different delivery technologies is expected to achieve efficient and safe delivery of nucleic acid drugs.

Develop pH/ATP dual-responsive lipid nanoparticles that can specifically release mRNA vaccines within tumor cells, increasing the uptake efficiency of antigen-presenting cells by 3 times in a lung cancer model. Fuse the deglycosylated antibody with a CTLA-4 inhibitor to form a bifunctional macromolecule, achieving the dual effects of increased T-cell infiltration and tumor metabolism inhibition in an esophageal cancer model, with a complete remission rate of 40%.

Fusion proteins combining antibody fragments with cell-penetrating peptides (CPPs) represent another breakthrough. Anti-HER2 scFv fused to TAT peptide (a CPP) showed enhanced internalization into HER2+ breast cancer cells by 300% compared to scFv alone, overcoming poor membrane permeability. Similarly, exosome-based carriers engineered to express tumor-targeting peptides (e.g., RGD for integrin-positive tumors) and loaded with ADCs achieved 4-fold higher tumor uptake than non-targeted exosomes in NSCLC models, leveraging exosomes’ natural ability to cross biological barriers and evade immune detection.

### 6.2 Combination therapy strategies

ICIs combined with chemotherapy, radiotherapy, or targeted therapy synergize to eliminate tumors. Radiotherapy enhances antigen release, while ICIs modulate the TME to sensitize tumors to radiation, reducing adverse reactions.

### 6.3 Development of personalized medicine

High-throughput sequencing, proteomics, and other techniques can be used to screen biomarkers for monitoring the efficacy of novel macromolecular drugs. For example, high-throughput sequencing can rapidly detect gene mutations in tumor cells, while proteomics can analyze protein expression and modifications. Integrating data such as tumor mutation burden and PD-L1 expression, and tumor mutation burden can predict response to immunotherapies, allow patient stratification, and provide guidance for personalized treatment. By leveraging AI and big data technologies, it is possible to analyze the clinical data, genetic information, and treatment responses of patients, and develop personalized treatment regimens for each patient. AI algorithms can process large amounts of data quickly, and assist doctors in selecting the most suitable drugs and dosages. Furthermore, machine learning algorithms can be used for constructing models to predict patients’ responses to different treatment regimens based on their clinical and genetic data. This provides decision-making support for doctors to formulate personalized treatment plans, and improve treatment efficacy and outcomes.

### 6.4 Market and social impact

The market share of novel macromolecular drugs will likely continue to expand in the coming years, as these drugs become mainstream for thoracic tumor treatment. Continuous innovations in immunotherapies and targeted therapies will further drive the demand for novel macromolecular drugs. Moreover, with the improvement of drug accessibility and reduction in costs, an increasing number of thoracic tumor patients will be able to afford these drugs. Nevertheless, the widespread use of novel macromolecular drugs poses higher requirements for the healthcare system, such as allocation of medical resources, establishment of specialized cancer treatment centers, and training the medical staff for patient evaluation, treatment, and monitoring. It is also necessary to adjust medical insurance policies to expand the reimbursement scope and increase the reimbursement ratio, thereby improving drug accessibility and reducing the economic burden on patients. Novel macromolecular drugs are expected to improve the survival rate and quality of life of patients with thoracic tumors, reduce the loss of labor force due to disease, and relieve the socio-economic burden. In addition, the research and development of these drugs would also drive biotechnological innovations, pharmaceutical manufacturing, and other industries, thereby generating employment opportunities and contributing to economic development.

Economic sustainability of these therapies demands cross-sector collaboration: payers must balance short-term costs against long-term savings from reduced hospitalizations and improved productivity. A cost-effectiveness analysis of pembrolizumab in NSCLC showed that despite a 3-year treatment cost of $150,000, it generated $85,000 in societal benefits via extended workforce participation. Policy levers such as mandatory price transparency, antitrust regulations to prevent monopolies, and public-private partnerships for joint R&D can drive down costs. In China, centralized drug procurement has reduced prices of 15 macromolecular oncology drugs by an average of 54% since 2021, significantly expanding patient access.

## 7 Conclusion

Novel macromolecular drugs have unique advantages due to their mechanisms of actions, and have demonstrated excellent clinical efficacy in the treatment of thoracic tumors. However, there are still numerous issues in the development, clinical application, and regulatory ethics of these drugs. Continuous technological innovation and interdisciplinary collaboration will be required to overcome these limitations, and develop more effective drugs to improve patient survival and quality of life. Furthermore, effective cooperation and sharing of resources and data among scientific research institutions, pharmaceutical companies, medical institutions, and regulatory authorities can accelerate the development and clinical translation of novel macromolecular drugs for thoracic tumors.

To address drug resistance, future research should focus on elucidating resistance mechanisms via multi-omics analysis, and develop combinatorial strategies such as co-targeting resistant pathways. For clinical challenges like tumor heterogeneity, personalized treatment guided by predictive biomarkers needs validation in large-scale trials. Additionally, optimizing delivery systems and reducing production costs through innovative manufacturing are critical for broadening accessibility. Regulatory frameworks should adapt to expedite approval of breakthrough therapies while ensuring safety, with ethical guidelines updated to address gene-editing related concerns in nucleic acid drugs.
